# Trans-Plasma Membrane Electron Transport and Ascorbate Efflux by Skeletal Muscle

**DOI:** 10.3390/antiox6040089

**Published:** 2017-11-09

**Authors:** Amanda M. Eccardt, Thomas P. Bell, Lyn Mattathil, Rohan Prasad, Shannon C. Kelly, Jonathan S. Fisher

**Affiliations:** 1 Department of Biology, Saint Louis University, 3507 Laclede Ave, St. Louis, MO 63103, USA; amanda.eccardt@slu.edu (A.M.E.); thomas.bell@slu.edu (T.P.B.); lyn.mattathil@slu.edu (L.M.); shannon.kelly@slu.edu (S.C.K.); 2 School of Medicine, Saint Louis University, 3507 Laclede Ave, St. Louis, MO 63103, USA; rohan.prasad@health.slu.edu

**Keywords:** tPMET, ascorbate, skeletal muscle, WST-1, GLUT1

## Abstract

Trans-plasma membrane electron transport (tPMET) and the antioxidant roles of ascorbate reportedly play a role in protection of cells from damage by reactive oxygen species, which have been implicated in causing metabolic dysfunction such as insulin resistance. Skeletal muscle comprises the largest whole-body organ fraction suggesting a potential role of tPMET and ascorbate export as a major source of extracellular antioxidant. We hypothesized that skeletal muscle is capable of tPMET and ascorbate efflux. To measure these processes, we assayed the ability of cultured muscle cells, satellite cells, and isolated extensor digitorum longus (EDL) and soleus (SOL) to reduce two extracellular electron acceptors, water soluble tetrazolium salt 1 (WST-1), and dichlorophenolindophenol (DPIP). Ascorbate oxidase (AO) was utilized to determine which portion of WST-1 reduction was dependent on ascorbate efflux. We found that muscle cells can reduce extracellular electron acceptors. In C2C12 myotubes and satellite cells, a substantial portion of this reduction was dependent on ascorbate. In myotubes, glucose transporter 1 (GLUT1) inhibitors along with a pan-GLUT inhibitor suppressed tPMET and ascorbate efflux, while a GLUT4 inhibitor had no effect. The adenosine 5′-monophosphate (AMP)-activated protein kinase activator 5-Aminoimidazole-4-carboxamide ribonucleotide (AICAR) suppressed both tPMET and ascorbate efflux by myotubes, while insulin had no effect. Taken together, our data suggest that muscle cells are capable of tPMET and ascorbate efflux supported by GLUT1, thus illustrating a model in which resting muscle exports electrons and antioxidant to the extracellular environment.

## 1. Introduction

In trans-plasma membrane electron transport (tPMET), electrons from cytosolic donors cross the plasma membrane, resulting in the reduction of extracellular oxidants; this process can occur via shuttle-based electron transport or be mediated by enzyme activity [1,2,3]. tPMET has been shown in a variety of cell types, including epithelial and smooth muscle cells, neurons, and erythrocytes [4]. In endothelial cells, tPMET activity reduces extracellular substrates. It does this by using electrons from intracellular reduced nicotinamide adenine dinucleotide (NADH) to blood-borne electron acceptors, resulting in an influence on vascular and organ function, as well as blood composition [4]. tPMET aids in maintaining redox homeostasis [5], meaning a balance between an oxidative environment and a reducing environment. This is beneficial, because it has been reported that an unbalanced redox state has been implicated as one of the primary factors leading to obesity-associated complications including types of metabolic dysfunction such as diabetes [6].

Ascorbate, more commonly known as vitamin C, is a water-soluble antioxidant. It has the capability to defend against oxidative stress and protect against lipid peroxidative damage by scavenging reactive oxygen species [7] in skeletal muscle [8,9]. A mechanism of ascorbate recycling has been illustrated in various cell types including astrocytes and hepatocytes [10,11]. The ascorbate recycling process involves export of ascorbate via gap-junctions, volume-sensitive (VSOAC) and Ca^2+^-dependent anion channels, exocytosis of secretory vesicles, and/or through plasma membrane hetero-exchange systems. Extracellular ascorbate is then oxidized to dehydroascorbic acid (DHA), which is taken back into the cells via glucose transporters (GLUTs) and reduced back into ascorbate [11]. 

In skeletal muscle, glucose transport is regulated by GLUT1 and GLUT4 [12,13,14,15,16,17]. GLUT1 and GLUT4 also transport dehydroascorbic acid (DHA), the oxidized form of ascorbic acid. GLUT1 is expressed in all tissue types and is localized primarily to the plasma membrane. Therefore, it mediates basal glucose transport in various cell types, including skeletal muscle cells [16,17]. GLUT4 is expressed at high levels in skeletal muscle and is the primary means of insulin-stimulated glucose uptake [12,13,14,15,17] and also insulin-independent glucose transport stimulated by factors such as metabolic stress [12,13,15,18]. GLUT1 and GLUT4 are the two isoforms of glucose transporters expressed in C2C12 myotubes [17,19]. It has been widely established that the release of insulin stimulates glucose transporter localization at the plasma membrane [12,14,20]. Insulin also stimulates DHA uptake by rat adipocytes and Xenopus oocytes expressing GLUT4 [21]. Intriguingly, GLUT1, but not GLUT4, appears to play a role in regulation of ROS levels in muscle cells [22].

Due to its large mass, skeletal muscle plays a key role in whole-body metabolism, and, if capable of tPMET, could play a predominant role in the extracellular redox environment. However, tPMET has not previously been characterized in skeletal muscle. Skeletal muscle contains about 40% of whole-body ascorbate [9]. Thus, if it is capable of exporting ascorbate, it could be substantial provider of extracellular antioxidant. We undertook this study to answer whether skeletal muscle cells are capable of tPMET and whether this process is dependent in part on ascorbate efflux. Further, given the roles of glucose transporters in uptake of DHA, we hypothesized that glucose transporters would play a role in support of tPMET and ascorbate efflux. Given the role of GLUT1 in regulation of ROS [22], we hypothesized that GLUT1 would play a key role in support of both tPMET and ascorbate efflux. Finally, we hypothesized that factors that regulate glucose transporters, such as insulin and the adenosine analog AICAR, would in turn influence tPMET and ascorbate efflux.

## 2. Materials and Methods

### 2.1. Materials

Phosphate buffered saline (PBS), trypsin- ethylenediamine tetraacetic acid, penicillin-streptomycin, and Dulbecco’s modified Eagle’s medium (DMEM) were purchased from Sigma Aldrich (St. Louis, MO, USA). FetalPlex was obtained from Gemini Bio-Products (Woodland, CA, USA). Horse serum was purchased from Gibco Technologies (Gaithersburg, MD, USA). 2,6-dichloroindophenol sodium salt (DPIP) was obtained from ICN Biomedicals Inc. (Aurora, OH, USA). Water-soluble tetrazolium salt 1 (WST-1) was obtained from Accela (San Diego, CA, USA). Phenazine methosulfate (PMS) was purchased from Acros Organics (Morris Plains, NJ, USA). D-glucose, phloretin, fasentin, STF-31, indinavir, and cytochalasin B were acquired from Sigma Aldrich (St. Louis, MO, USA). 5-Aminoimidazole-4-carboxamide-1-β-d-ribofuranoside (AICAR) was purchased from Toronto Research Chemicals (Toronto, ON, Canada). Goat anti-mouse and goat anti-rabbit secondary antibodies conjugated to horseradish peroxidase were obtained from Thermo Scientific (Rockford, IL, USA).

### 2.2. Animals

C57 Black 6 mice were obtained from the Jackson Laboratory (Bar Harbor, ME, USA). The animals had open access to food and water. Additionally, the mice were kept in a temperature-controlled environment which consisted of a 12 h light-dark cycle. Pentobarbital (50 mg/kg, IP) was used as the form of anesthesia. After anesthesia administration, soleus (SOL) and extensor digitorum longus (EDL) were extracted and immediately used for experimentation. Animals were killed while under anesthesia by thoracotomy. Each procedure utilizing animals was approved by the Saint Louis University Institutional Animal Care and Use Committee (IACUC approval number 2453).

### 2.3. Cell Culture 

C2C12 myoblasts were bought from the American Type Culture Collection (Manassas, VA, USA). Primary myoblasts obtained from mouse gastrocnemius-soleus complexes were generously provided by Koyal Garg (Saint Louis University, St. Louis, MO, USA). DMEM containing 1% penicillin-streptomycin solution and 10% FetalPlex were utilized to culture the C2C12 myoblasts in 5% CO_2_ at 35 °C [23]. Cells were plated in 96-well plates. Once ~70% confluence was attained, the myoblasts were differentiated into myotubes in a solution of DMEM containing 2% horse serum and 1% penicillin-streptomycin.

### 2.4. DPIP Reduction Assays

To monitor tPMET by mouse SOL and EDL, DPIP was used as the extracellular electron acceptor [24]. After tissue extraction, muscles were recovered in a Krebs-Henseleit buffer (KHB) containing 8 mM glucose and 32 mM mannitol (Sigma Aldrich, St. Louis, MO, USA) at 35 °C with a gas phase of 95%:5% (O_2_:CO_2_) for 20 min. Tissues were then incubated in 2 mL of a reaction medium containing 100 µM DPIP in recovery solution for 1 h at 35 °C. 100 µL of the reaction media was taken from each well every 10 min (over the 1 h incubation) in order to read the absorbance at 620 nm. Wells containing only reaction medium and no muscle tissue were used to account for background. After completion of the assay, the muscles were transferred to fresh recovery solution and incubated for 20 min following the parameters previously mentioned. The muscles were then trimmed, frozen via freeze clamps in liquid nitrogen, and homogenized using lysis buffer (50 mM 4-(2-hydroxyethyl)-1-piperazineethanesulfonic acid (HEPES) pH 7.4, 150 mM NaCl, 10% glycerol, 1.5 mM MgCl, 1 mM EDTA, 10 mM NaPO_4_, 100 mM NaF, 2 mM NaVO_4_, 10 mg/mL leupeptin, 10 mg/mL aprotinin, 0.5 mg/mL pepstatin, and 1 mM phenylmethylsulfonyl fluoride) and 0.5 mm zirconium oxide beads (MidSci, St. Louis, MO, USA) in a Bullet Blender (Next Advance, Averill Park, NY, USA). Once homogenized, 1% Triton X-100 was added to each sample. The muscle homogenate was used in a bicinchoninic acid (BCA) protein assay (Thermo Scientific, Rockford, IL, USA) in order to quantify data based on protein content. Nanomoles of DPIP reduced for each time period were calculated using an extinction coefficient for oxidized DPIP of 21 mM^−1^ cm^−1^ [25] and adjusted for the volume of assay reagent remaining at each time point.

### 2.5. WST-1 Reduction Assays Monitoring tPMET and Ascorbate Efflux

To characterize tPMET by cultured C2C12 myotubes and primary myotubes, WST-1 was used as the membrane impermeable extracellular electron acceptor, as has been previously described [24,26]. For all experiments utilizing WST-1, the reaction media consisted of 400 µM WST-1, 20 µM PMS, and 5 mM glucose in PBS. For all experiments, the absorbance was read every 10 min for 1 h at 438 nm using a PowerWave X-I plate reader (BioTek, Winooski, VT, USA). Wells containing only reaction medium and no C2C12 or primary myotubes were used to account for background. Following completion of the 1 h reading, the wells were washed twice with PBS. A BCA protein assay was then performed in order to quantify samples based on protein content.

In order to first assess the capability of myotubes to participate in tPMET, myotubes were treated with 100 µM ascorbic acid (ICN Biomedicals Inc., Aurora, OH, USA) overnight in 5% CO_2_ at 35 °C. The cells were incubated in the reaction media in the presence/absence of 2 U/mL ascorbate oxidase (Sigma Aldrich, St. Louis, MO, USA) to determine whether ascorbate efflux contributed to tPMET by muscle cells.

### 2.6. WST-1 Reduction Assays Monitoring GLUT1 Involvement in tPMET and Ascorbate Efflux

GLUT1 involvement in tPMET was determined by exposing the cells to a variety of glucose transport inhibitors (100 µM phloretin, 80 µM fasentin, 5 µM STF-31, 10 µM cytochalasin B, and 100 µM indinavir). The experiment consisted of a 30 min pretreatment in the presence or absence of the inhibitor in DMEM or PBS supplemented with 5 mM glucose at 35 °C. The same cells pretreated with the inhibitor also had the inhibitor present in the WST-1 reaction media.

### 2.7. FLAG-GLUT1 Transfections and GLUT1 Involvement in tPMET

Myoblasts were transfected with a FLAG-GLUT1 construct [27] provided by Jeffrey Rathmell (Duke University, Durham, NC, USA) using lipofectamine 2000 (Invitrogen, Carlsbad, CA, USA) following the manufacturer’s instructions. One day after transfection, the cells were subjected to WST-1 reduction assays or washed twice with PBS and harvested on ice using lysis buffer (50 mM HEPES pH 7.4, 150 mM NaCl, 10% glycerol, 1% Triton X-100, 1.5 mM MgCl_2_, 1 mM EDTA, 10 mM, sodium pyrophosphate, 100 mM NaF, 2 mM Na_3_VO_4_, 10 µg/mL aprotinin, 10 µg/mL leupeptin, 0.5 µg/mL pepstatin, and 0.2 mM phenylmethylsulfonyl fluoride). Whole cell homogenate protein content was then quantified using a BCA protein assay. Samples were combined with Laemmli sample buffer and were run on 4–20% pre-cast RunBlue sodium dodecyl sulfate (SDS) gels (Expedeon, Inc., San Diego, CA, USA) and transferred to nitrocellulose membranes (Thermo Scientific, Rockford, IL, USA). The membranes were then blocked in 5% nonfat dry milk in tris-buffered saline plus 0.1% Tween (TBST) pH 7.4. The membranes then incubated in anti-FLAG or anti-GLUT1 primary antibodies. The antibody against GLUT1 was kindly provided by Michael Mueckler (Washington University School of Medicine, St. Louis, MO, USA). Following primary antibody incubation, the membranes were washed in TBST and incubated with horseradish peroxidase (HRP)-linked goat-anti-mouse or goat-anti-rabbit secondary antibodies. Membranes were then washed using TBST and TBS, and bands were detected using enhanced chemiluminescence (PerkinElmer Life Sciences, Boston, MA, USA). Following initial imaging, membranes were washed with TBST and incubated with HRP-conjugated antibodies against glyceraldehyde 3-phosphate dehydrogenase (GAPDH-HRP) (Cell Signaling Technology, Danvers, MA, USA). GLUT1 bands were then normalized to GAPDH bands using the computer program Total LabQuant software (Cleaver Scientific, Warwickshire, UK).

Following the protocol previously stated, WST-1 assays were also utilized post transfection (described above) in order to determine if increasing GLUT1 expression had an effect on tPMET. 

### 2.8. WST-1 Reduction Assays Monitoring Effects of Glucose on tPMET

In order to determine if tPMET was a glucose dependent process, C2C12 myotubes were incubated in the reaction media above with the following glucose concentrations: 0 mM, 5 mM, 10 mM, and 15 mM. 2 mM sodium pyruvate (Sigma Aldrich, St. Louis, MO, USA) replaced glucose in the 0 mM concentration. 

### 2.9. WST-1 Reduction Assays Monitoring Effects of AICAR and Insulin on tPMET and Ascorbate Efflux

To observe how factors that affect metabolism contribute to tPMET, C2C12 myotubes were incubated in media containing the presence or absence of 2 mM AICAR or 100 nM insulin. The assays that utilized AICAR also had a 100 µM ascorbic acid pretreatment. As previously mentioned, the reaction media consisted of the presence or absence of 2 U/mL ascorbate oxidase and 2 mM AICAR. The assay that utilized insulin consisted of a 30 min pretreatment in the presence or absence of 100 nM insulin. The extinction coefficient used for reduced WST-1 was 37 mM^−1^ cm^−1^ [28].

To determine if AICAR or insulin had an effect on glucose-6-phasphate dehydrogenase (G6PD), an activity assay was completed following previous studies by Passonneau and Lowry [29]. As mentioned above, C2C12 myotubes were pretreated in the presence or absence of 2 mM AICAR or 100 nM insulin. The G6PD activity reagent consisted of 100 mM 2-amino-2-methyl-1-propanol, pH 9.4, 50 μM NADP^+^, 0.5 mM EDTA, and 0.02% bovine serum albumin (BSA) [30]. The assay began by incubating in the presence or absence of 1 mM glucose 6-phosphate (G6P). The reaction was then monitored spectrophotometrically by an increase in absorbance at 340 nm corresponding to an increase in reduced nicotinamide adenine dinucleotide phosphate (NADPH). 

## 3. Results

### 3.1. C2C12 Myotubes, Primary Myotubes, and Isolated Mouse SOL and EDL Are Capable of tPMET

**Figure 1:**
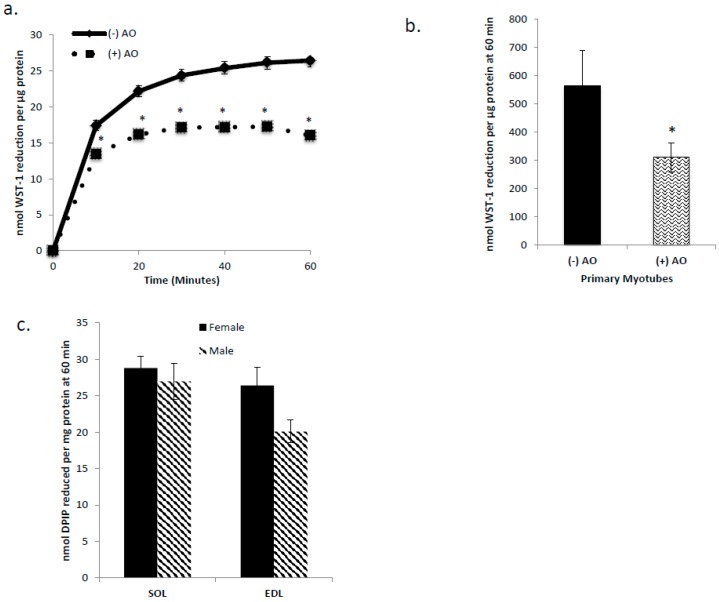
Cultured myotubes, primary myotubes, and isolated mouse soleus (SOL) and extensor digitorum longus (EDL) are capable of trans-plasma membrane electron transport (tPMET). (**a**) WST-1 reduction by cultured myotubes in the presence or absence of ascorbate oxidase (AO). A decrease in WST-1 reduction in the presence of AO indicates that a portion of tPMET is attributable to the export of ascorbate. *N* = 18/group, * *p* < 0.05 (**b**) WST-1 reduction by primary myotubes in the presence or absence of ascorbate oxidase. *N* = 15, * *p* < 0.05 (**c**) DPIP reduction by mouse EDL and SOL. Female: *N* = 6, *p* = 0.404 between female SOL and EDL. Male: *N* = 3, *p* = 0.096 between male SOL and EDL. *p* = 0.075 between female and male EDL. *p* = 0.584 between female and male SOL.

In order to determine if C2C12 myotubes, primary myotubes, and isolated mouse SOL and EDL were capable of tPMET, the membrane impermeable electron acceptors WST-1 and DPIP were used. Cultured myotubes and primary myotubes incubated in the presence or absence of ascorbate oxidase in order to determine the involvement of ascorbate in tPMET. The WST-1 reduction by cultured myotubes shown in Figure 1A demonstrates that muscle cells are capable of tPMET. In the presence of ascorbate oxidase, WST-1 reduction was suppressed by approximately 40%, indicating that 40% of tPMET in cultured myotubes is attributable to ascorbate efflux (*p* < 0.05 Figure 1a). As shown in Figure 1b, primary myotubes display a ~20-fold higher tPMET than C2C12 myotubes. However, as in C2C12 myotubes, AO suppressed WST-1 reduction by about 40% in the primary myotubes. As shown in Figure 1c, both SOL and EDL muscles from mice are capable of tPMET. There is a statistically non-significant trend for a greater capability of tPMET by male SOL versus EDL (*p* = 0.096) and by female EDL versus male EDL (*p* = 0.075).

### 3.2. GLUT1 is the Primary Glucose Transporter Involved in tPMET

**Figure 2:**
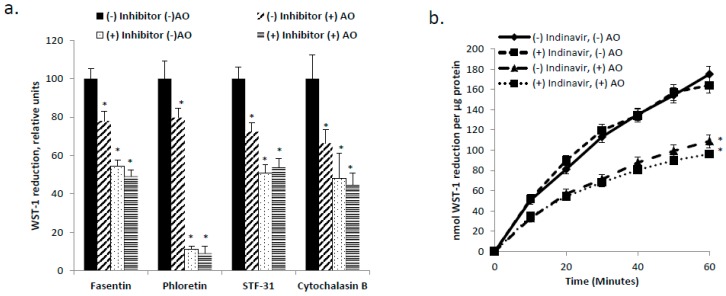
Glucose transporter 1 (GLUT1) supports tPMET. (**a**) In the presence of the GLUT1 inhibitors, fasentin, phloretin, and STF-31, and the inhibitor of all GLUTs, cytochalasin B, tPMET is decreased by C2C12 myotubes. *N* = 18/group, * *p* < 0.05 versus (−) inhibitor (−) AO (**b**) In the presence of the GLUT4 inhibitor, indinavir, there is no effect on tPMET. *N* = 18/group, * *p* < 0.05 versus corresponding group without AO.

GLUT1 and GLUT4 are the primary glucose transporters present in skeletal muscle [12] and in C2C12 myotubes [17,19]. Given reports that GLUT1 and GLUT4 are DHA transporters [21,31], as well as the central role of DHA uptake in the ascorbate cycling previously described for hepatocytes [10,11], we hypothesized that inhibition of the GLUTs would impede tPMET and ascorbate efflux. As shown in Figure 2a, in the presence of the GLUT1 inhibitors, phloretin, fasentin, and STF-31, and the pan-GLUT inhibitor, cytochalasin B, tPMET is suppressed in C2C12 myotubes (*p* < 0.05). However, AO sensitivity of WST-1 reduction was completely abolished by the GLUT1 inhibitors (Figure 2a), indicating that inhibition of GLUT1 prevented ascorbate efflux. The GLUT4 inhibitor, indinavir, had no effect on tPMET by C2C12 myotubes (*p* < 0.05 Figure 2b). Taken together, these data suggest that GLUT1 is the primary GLUT involved in supporting tPMET. 

### 3.3. Increasing GLUT1 Expression Increases tPMET

**Figure 3:**
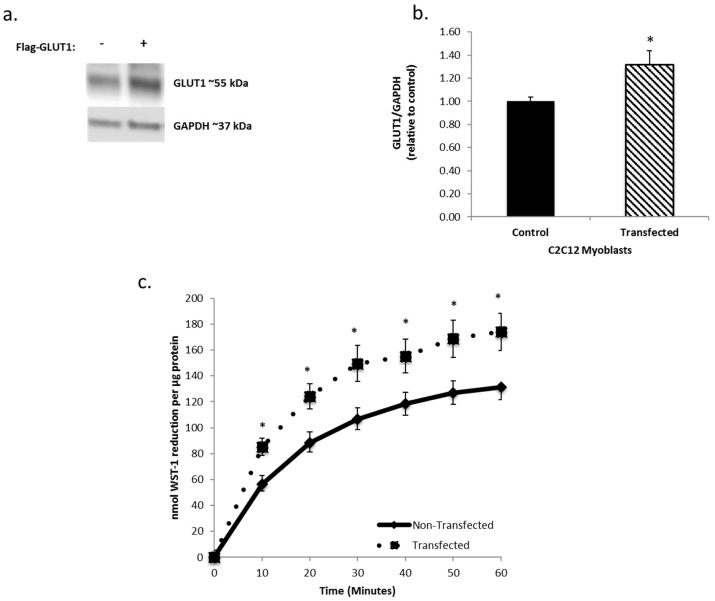
Increasing GLUT1 expression increases trans-plasma membrane electron transport (tPMET). (**a**) Western blot analysis confirms effective lipofectamine transfection. Glyceraldehyde 3-phosphate dehydrogenase (GAPDH) was utilized as the loading control. *N* = 3 (**b**) Western blot quantification demonstrates that the transfected samples have a ~30% increase in GLUT1 expression. *N* = 3/group, * *p* < 0.05 versus non-transfected. (**c**) C2C12 myoblasts transfected with FLAG-GLUT1 show a ~30% increase in tPMET. *N* = 18/group, * *p* < 0.05 versus non-transfected

To further investigate the role of GLUT1 in tPMET, C2C12 myoblasts were transfected with a FLAG-GLUT1 plasmid (Figure 3a). Transfected cells had ~30% more GLUT1 than non-transfected cells (Figure 3b). As shown in Figure 3c, increasing GLUT1 expression increased tPMET by ~30% by C2C12 myoblasts (*p* < 0.05), reinforcing that GLUT1 supports tPMET in C2C12 myotubes.

### 3.4. tPMET is a Glucose Dependent Process

**Figure 4:**
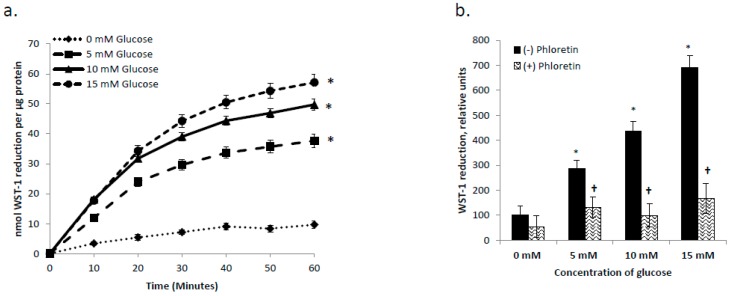
tPMET is a glucose-dependent process. (**a**) tPMET increased by C2C12 myotubes as glucose concentration increased. *N* = 18/group, * *p* < 0.05 versus 0 mM glucose. (**b**) tPMET increases as glucose concentration increases and is suppressed in the presence of phloretin. *N* = 7/group, * *p* < 0.05 versus 0 mM glucose. † *p* < 0.05 versus (−) inhibitor at that concentration.

Having established that GLUT1 plays a role in support of tPMET (Figure 2a and Figure 3c) and ascorbate efflux (Figure 2a), we then asked whether GLUT1’s influence was via mediation of glucose transport (as opposed to DHA uptake). As shown in Figure 4a, increasing concentrations of glucose increased tPMET by C2C12 myotubes (*p* < 0.05). At 0 mM glucose, tPMET is essentially completely suppressed, indicating that tPMET by C2C12 myotubes is a glucose-dependent process (Figure 4a). As shown in Figure 4b, as glucose concentration increases, tPMET by C2C12 myotubes increases. However, in the presence of the GLUT1 inhibitor phloretin, tPMET is suppressed amongst all concentrations of glucose excluding 0 mM (*p* < 0.05 Figure 4b).

### 3.5. Effects of Insulin and AICAR on tPMET

**Figure 5:**
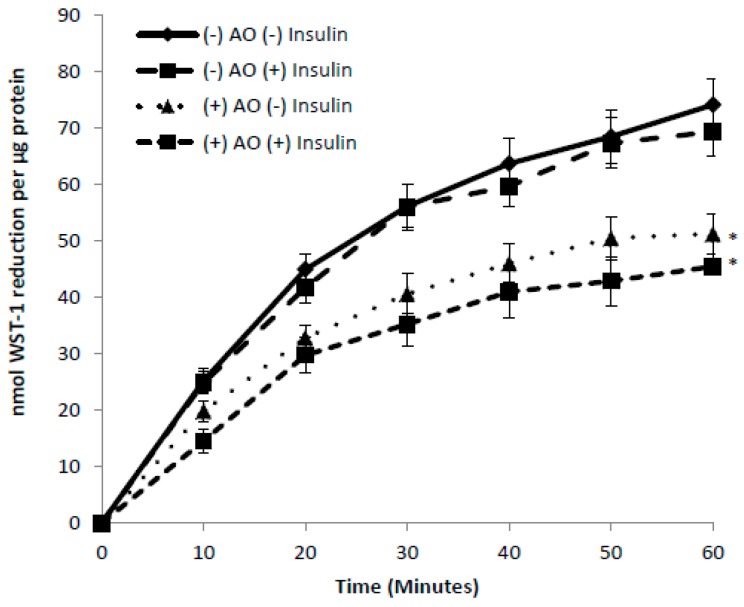
Insulin has no effect on tPMET in C2C12 myotubes. Myotubes were incubated in the absence or presence of insulin before assay of tPMET. *N* = 18/group, * *p* < 0.05 versus (−) insulin (−) AO.

**Figure 6:**
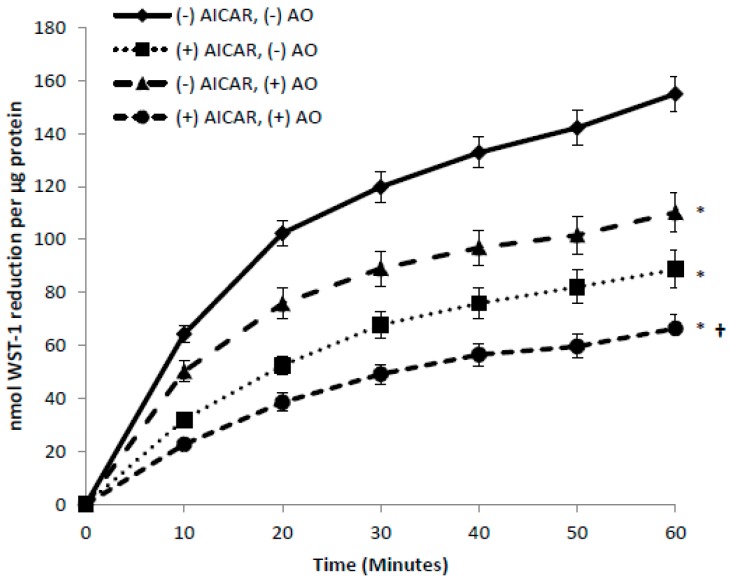
5-Aminoimidazole-4-carboxamide ribonucleotide (AICAR) suppresses tPMET in C2C12 myotubes. Myotubes were incubated in the absence or presence of AICAR before tPMET assays. *N* = 18/group, * *p* < 0.05 versus (−) AICAR (−) AO, † *p* < 0.05 versus (+) AICAR, (−) AO.

As demonstrated above, GLUT1 appears to be the GLUT that supports tPMET under basal conditions. GLUT1 is responsible for the majority of basal glucose transport in skeletal muscle [16,17,32]. In contrast, GLUT4 translocation to the cell surface is the primary means for increased glucose transporter action in response to factors such as insulin and AICAR, an activator of the AMP-activated protein kinase [33,34,35]. Thus, we asked whether under conditions that would increase GLUT4-mediated transport (i.e., incubation with insulin or AICAR) would also increase tPMET. However, exposing C2C12 myotubes to insulin had no effect on tPMET (Figure 5). We next examined whether AICAR would influence tPMET. AICAR, an adenosine analog, is taken up by cells and phosphorylated to the AMP analog AICA-ribotide, which activates AMP-activated protein kinase (AMPK), thus providing a model of metabolic stress [36]. Interestingly, AICAR caused a suppression of tPMET by C2C12 myotubes (*p* < 0.05 Figure 6), suggesting a role of AMPK in this process. There was also a trend for a decrease in the AO-sensitive portion of tPMET, suggesting that AICAR also decreases ascorbate efflux. We then assessed if observed effects on tPMET by treatment of insulin or AICAR were due to inhibition of glucose-6-phosphate dehydrogenase. A G6PD assay was completed, which monitors the ability of glucose-6-phosphate dehydrogenase to catalyze the oxidation of glucose-6-phosphate to 6-phospho-D-glucono-1,5-lactone. There was no effect of insulin or AICAR on G6PD activity (control: 156.9 ± 27.8 nmol/µg protein/min, insulin: 154.9 ± 9.8, AICAR: 155.3 ± 7.95). 

## 4. Discussion

The new evidence found by this study illustrates that skeletal muscle cells are capable of tPMET, partially through export of ascorbate (Figure 1a,b). Additionally, GLUT1 is the primary glucose transporter involved in tPMET by C2C12 myotubes (Figure 2a,b and Figure 4b), while increasing GLUT1 expression increases tPMET (Figure 3a–c). Through this study, it was determined that tPMET by C2C12 myotubes is a glucose-dependent process (Figure 4a,b). In the presence of the AMPK-activator, AICAR, tPMET and ascorbate efflux is suppressed (Figure 6). However, the addition of insulin had no effect on tPMET or ascorbate efflux (Figure 5). 

Previous studies suggest that tPMET plays expansive roles in maintenance of multiple physiological functions. For instance, a primary function of tPMET is to aid in maintenance of the cytoplasmic NAD^+^/NADH ratio, which has been shown to regulate metabolic enzymes such as the pyruvate dehydrogenase complex and glyceraldehyde-3-phosphate dehydrogenase [5,37,38,39]. It has been presented that under states of mitochondrial dysfunction such as diabetes mellitus [5,40], consistent hyperglycemia yields an abundance of NADH causing a redox imbalance of the NAD^+^/NADH ratio [41]. Thus, it is plausible that tPMET has the capability to regulate the maintenance of this ratio [5] under such dysfunction. The activity of tPMET is also very important in maintaining blood redox; for example, it has been shown that tPMET is correlated to the metabolic status of erythrocytes [24,42].

As well as physiological functions, tPMET has been shown to take on pathological roles including influencing apoptosis. Apoptosis is a strictly monitored form of cell death involved in tissue development and maintenance of body homeostasis [5]. Lack of apoptosis can lead to excessive cell proliferation and cancer, while extreme apoptosis can lead to hypotrophy [5]. As mentioned above, tPMET maintains a balanced redox state. An imbalance in tPMET leading to a pro-oxidant environment at the plasma membrane can induce unwarranted apoptosis [43].

Previously mentioned, GLUT1 and GLUT4 regulate glucose transport in skeletal muscle [12,13,14,15,16,17] and also transport DHA. GLUT1 localization, primarily at the plasma membrane, suggests a main role of mediation of basal glucose transport [16,17]. In contrast, GLUT4 is primarily responsible for insulin-stimulated glucose uptake [12,13,14,15,17]. This study has shown that GLUT1, but not GLUT4, supports tPMET and ascorbate efflux (Figure 2a,b). Inhibition of GLUT1 leads to a suppression of tPMET and ascorbate efflux Figure 2a and Figure 4b), while increasing GLUT1 expression resulted in an increase in tPMET (Figure 3c). Such evidence is consistent with multiple potential roles of GLUT1 in regard to tPMET, including enhanced glucose and DHA transport and possible influence on pentose phosphate pathway (PPP) activity. Glucose enters the cell and is almost immediately phosphorylated by hexokinase to glucose-6-phosphate, a main player in the PPP [44]. As is shown, increasing glucose concentration increases tPMET (Figure 4a,b), thus suggesting a possible role and increased flux of the PPP and involvement in tPMET. 

AICAR, an AMPK activator, suppressed tPMET and ascorbate efflux (Figure 6). We speculated that this could be due to inhibition of the PPP. However, as mentioned above, AICAR had no effect on G6PD activity. However, suppression of tPMET and ascorbate efflux may coincide with the energy-conserving actions of AMPK [45]. 

## 5. Conclusions

Overall, this study has demonstrated that skeletal muscle cells, which comprise the largest whole-body organ fraction, are capable of tPMET and ascorbate efflux in a manner dependent on GLUT1 and glucose. Thus, taken together, these data suggest that tPMET and ascorbate export could be manipulated to either preserve the intracellular ascorbate pool or maximize extracellular antioxidant status.
